# Robust imaging habitat computation using voxel-wise radiomics features

**DOI:** 10.1038/s41598-021-99701-2

**Published:** 2021-10-11

**Authors:** Kinga Bernatowicz, Francesco Grussu, Marta Ligero, Alonso Garcia, Eric Delgado, Raquel Perez-Lopez

**Affiliations:** 1 Radiomics Group, Vall d’Hebron Institute of Oncology (VHIO), 08035 Barcelona, Spain; 2 Radiology Department, Vall d’Hebron University Hospital, 08035 Barcelona, Spain

**Keywords:** Biomedical engineering, Cancer imaging

## Abstract

Tumor heterogeneity has been postulated as a hallmark of treatment resistance and a cure constraint in cancer patients. Conventional quantitative medical imaging (radiomics) can be extended to computing voxel-wise features and aggregating tumor subregions with similar radiological phenotypes (imaging habitats) to elucidate the distribution of tumor heterogeneity within and among tumors. Despite the promising applications of imaging habitats, they may be affected by variability of radiomics features, preventing comparison and generalization of imaging habitats techniques. We performed a comprehensive repeatability and reproducibility analysis of voxel-wise radiomics features in more than 500 lung cancer patients with computed tomography (CT) images and demonstrated the effect of voxel-wise radiomics variability on imaging habitats computation in 30 lung cancer patients with test–retest images. Repeatable voxel-wise features characterized texture heterogeneity and were reproducible regardless of the applied feature extraction parameters. Imaging habitats computed using robust radiomics features were more stable than those computed using all features in test–retest CTs from the same patient. Nine voxel-wise radiomics features (joint energy, joint entropy, sum entropy, maximum probability, difference entropy, Imc1, Imc2, Idn and Idmn) were repeatable and reproducible. This supports their application for computing imaging habitats in lung tumors towards the discovery of previously unseen tumor heterogeneity and the development of novel non-invasive imaging biomarkers for precision medicine.

## Introduction

Intra-tumor spatial heterogeneity, due to diverse tumor clonality and the microenvironment has been thought to drive tumor adaptation and resistance over the course of treatment in cancer patients^1^. This may have severe clinical implications, such as rapid tumor progression and short survival^2,3^. Accurate characterization of the spatio-temporal landscape of an individual patient’s tumor phenotype is critical for providing guidance in personalized treatment.

Noninvasive medical imaging enables longitudinal quantification of tissue properties. Quantitative medical imaging (radiomics) provides an excellent tool to characterize cancer cells and the tumor microenvironment, and quantify changes following therapy. The field of radiomics has been well established over the last decade, exploiting a large set of image features extracted from segmented volumes-of-interest (VOI) to build several classifier models allowing identification of distinct groups with different biology or to predict the clinical outcome^4–7^.

Conventional radiomics analysis allows for quantification of multiple tumor features, including shape, first-order (histogram-based), and second-order (texture-based) characteristics. Second-order features can be extracted from 3D volume, however ultimately this information is collapsed to 1D corresponding to a single VOI statistic e.g., mean homogeneity. In this context, conventional radiomics assumes that the tumor volume can be represented by a combination of these VOI-wise, averaged statistics. Yet tumors are heterogeneous, presenting different tumor phenotypes between and within lesions; therefore, to better characterize the spatial distribution of these phenotypes, conventional radiomics analysis should be extended to 3D. This can be achieved by computing voxel-wise features and aggregating tumor subregions with similar radiological phenotypes (imaging habitats).

Imaging habitats extracted from computed tomography (CT) images reveal previously unseen heterogeneity. This has been shown to improve the classification accuracy of diagnostic and predictive models in ovarian^8^ and lung^9^ cancer patients. Moreover, CT habitats were recently used to select regions for ultrasound-guided biopsy in ovarian cancer patients^10^. However promising, clinical translation of the imaging habitats technique requires demonstrating robustness and validity of the generated radiomics models. The reproducibility of conventional radiomic features has been previously assessed in a number of publications^11–13^, but the voxel-wise (3D) feature reproducibility has not been explored, and this aspect may dramatically affect the imaging habitats computation.

To address this gap in knowledge, we conducted a comprehensive study to assess the repeatability and reproducibility of voxel-wise (3D) radiomic features in more than 500 lung cancer patients CT scans. In this study, we address five pressing questions: Is repeatability of 3D radiomics features the same as in conventional 1D radiomics features? Which 3D features are reproducible? How robust are 3D features to extraction parameters (i.e., bin width and kernel radius)? Can reproducibility be studied using image perturbation in cases where test–retest data are unavailable? Finally, what is the effect of robust features selection on computed imaging habitats?

## Results

### Repeatability and reproducibility of voxel-wise (3D) radiomics features in test–retest data

#### VOI-wise vs voxel-wise radiomics feature repeatability

**Figure 1:**
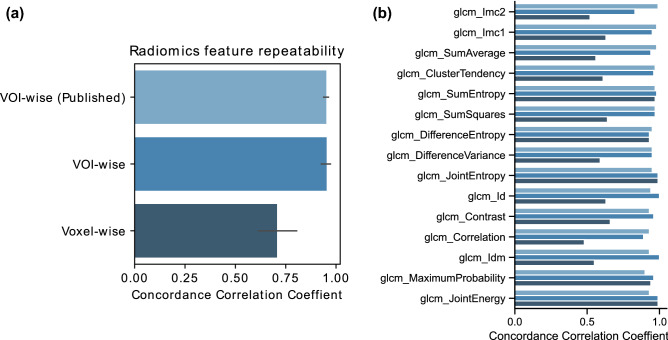
VOI-wise vs voxel-wise feature repeatability using the concordance correlation coefficient (CCC). (**a**) Volume-of-interest (VOI)-wise radiomics features were extracted from Zhao et al. (Supplementary Table S2) and also computed in this study (first two bars). Bar plot represents the average and standard deviation of the CCC values from all features extracted from all test–retest computed tomography (CT) scans. (**b**) Length of the bar represents the mean CCC value per analyzed feature across computed test–retest CT scans.

We observed high agreement between VOI-wise feature repeatability, as calculated both using data from a previously published study^14^, and from the current study using the same dataset, with median concordance correlation coefficients (CCC) of 0.95 and 0.96, respectively (Fig. 1). Small differences could be attributed to the differences in segmentation methods in the two studies. Here, test and retest CT images were first registered and the test lesions were rigidly aligned onto retest images for evaluation, whereas in the previously published study, two different segmentations were used, i.e., from test and retest image^14^. In contrast, voxel-wise feature repeatability was much lower than VOI-wise feature repeatability in our study, with a median CCC of 0.63.

#### Individual voxel-wise radiomics features repeatability

**Figure 2:**
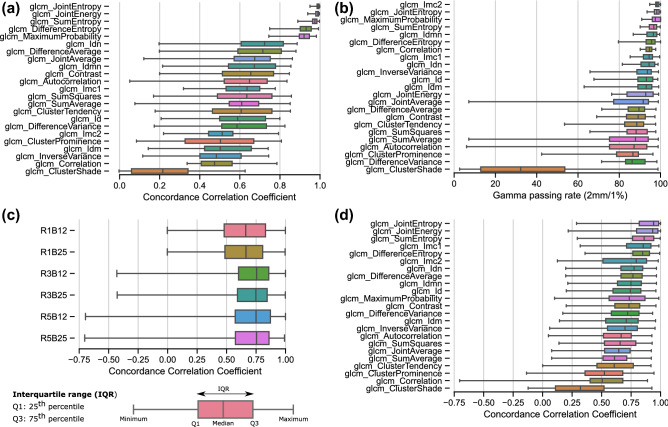
Repeatability and reproducibility of voxel-wise radiomics features in test–retest data. (**a**) Most repeatable features are located at the top of the graphic according to CCC and (**b**) gamma passing rate (Γ-index). The gamma (Γ) acceptance criterion (2 mm/1%) is defined as the distance to agreement (DTA) and the maximum feature value in percent (F_max_), as shown in the insert. Features were extracted with a fixed bin size of 12 and kernel radius of 1. (**c**) Reproducibility of voxel-wise radiomics features to feature extraction parameters (B = bin size and R = kernel radius). (**d**) Reproducibility of voxel-wise features computed using all combinations of feature extraction parameters.

Five out of 23 radiomics features explored had a median CCC > 0.9 in the evaluated patient lesions and were selected as repeatable (Fig. 2a). These included joint energy, joint entropy, sum entropy, maximum probability and difference entropy, which characterize texture heterogeneity. The gamma passing rate (Γ-index), based on 2 mm/1% acceptance criterion, resulted in increased repeatability estimation in voxel-wise features. Six features had a median gamma passing rate (defined as the percentage of voxels that passed the acceptance criteria Γ < 1), higher than 95% in the studied test–retest dataset and were defined as repeatable (Fig. 2b). Four of these six repeatable features were similar between CCC and gamma passing rate evaluations. The additional repeatable features were informational measure correlation (Imc2) and inverse difference momentum normalized (Idmn).

The repeatability of voxel-wise features using other metrics i.e., structural similarity index measure (SSIM) and Pearson’s correlation (PCORR) is provided in Supplementary Document SD7. In general, all metrics resulted in very similar feature repeatability ranking. Imc2 was an additional repeatable feature identified with SSIM, which is in agreement with the Γ-index finding.

#### Reproducibility of voxel-wise radiomics features to radius and bin size changes

The reproducibility of all voxel-wise features in test–retest data for different combinations of bin width (B) and kernel radius (R) is shown in Fig. 2c. The CCC value distribution was more sensitive to changes in R than to changes in B. The median CCC was highest when R = 3, but the interquartile range was smaller when R = 1. Although the distribution of median CCC values had changed (shifted towards lower values), the feature ranking seems independent of the feature extraction parameters; the top robust features were joint energy, joint entropy, sum entropy, difference entropy and maximum probability, as shown in Fig. 2d. Additionally, Imc1 and inverse difference normalized (Idn) could be considered robust to changes in bin size and radius.

From the nine identified repeatable and reproducible features (joint energy, joint entropy, sum entropy, maximum probability, difference entropy, Imc1, Imc2, Idn and Idmn), three structurally-different groups can be distinguished based on SSIM: (1) joint entropy, sum entropy, difference entropy, and Imc2 (2) maximum probability and joint energy, and (3) Idn, Idmn and Imc1, as demonstrated in Supplementary Document, SD8. These groups were confirmed with a positive PCORR between the features within the groups. Additionally, a negative correlation between groups 1 and 2 was found.

### Repeatability of voxel-wise features in test-perturbed data

#### Individual voxel-wise radiomics features repeatability

**Figure 3:**
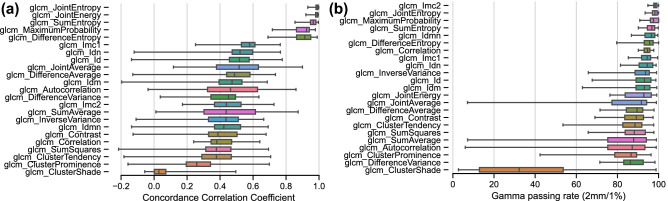
Repeatability of voxel features in test-perturbed data. Most repeatable features are located at the top of the graphic (**a**) according to the median CCC and (**b**) Γ-index. Features were extracted with a fixed bin size of 12 and kernel radius of 1.

Five out of 23 computed radiomics features had a median CCC > 0.9 as identified using perturbed images as a surrogate of retest images (compare Figs. 2a and 3a). Similar observations were true when the Γ-index was considered; all 6 repeatable features were identified (with median gamma passing rate above 93.4%) with only one feature discordantly classified as repeatable i.e., correlation (compare Figs. 2b and 3b).

The repeatability of voxel-wise features using other metrics (SSIM and PCORR) is provided in Supplementary Document SD7. All metrics result in a very similar feature ranking and the additional repeatable feature based on SSIM is Imc2, which is in agreement with the Γ-index finding and similar to the evaluation in test–retest data.

#### Repeatability of voxel-wise radiomics in other datasets

The repeatability of voxel-wise features (CCC) based on image perturbations was also assessed in two other datasets: “Radiomics-NSCLC” consisting of 422 CT scans and “VHIO dataset” of 60 CT images. Results are shown in Supplementary Document SD10. Although the magnitude of CCC differs between “RIDER” and other datasets, the feature ranking remains similar, supporting selection of entropy- and energy-based features for robust imaging habitats computation in lung tumors.

### Robustness of imaging habitats

**Figure 4:**
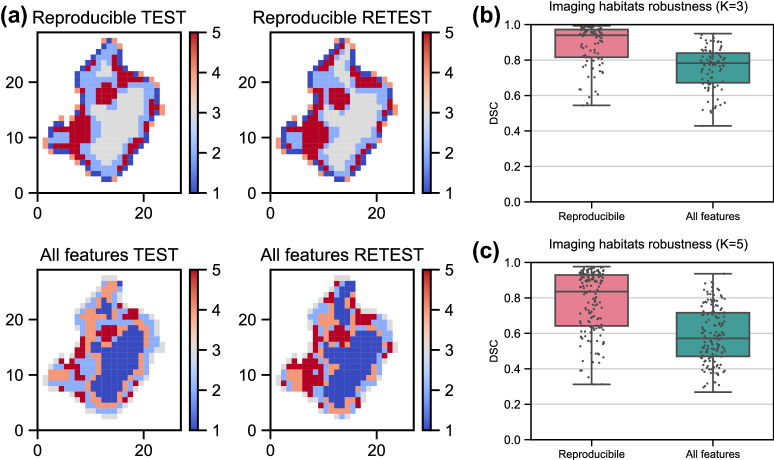
Robustness of imaging habitats to feature selection. (**a**) Axial section of imaging habitats computed using selected features (repeatable and reproducible; upper figures) and using all features (lower figures) in patient’s test and retest CTs. Five imaging habitats were computed in each case (see color bar). (**b**,**c**) Robustness of imaging habitats quantified using Dice Similarity Coefficient (DSC) between habitats computed in test and retest images of all 30 lung cancer patients. Different numbers of habitats per lesion were evaluated; K = 3 and K = 5. Observations are shown on top of a boxplot.

Finally, the effect of repeatable and reproducible feature selection on imaging habitats computation was analyzed in the RIDER data. Fig. 4 shows an example of imaging habitats (K = 5) computed based on voxel-wise features extracted from test–retest CTs. It is apparent that imaging habitats computed in test–retest CTs from the same patient using selected features resulted in more stable habitats than when all features were used in computation (Fig. 4a).

This observation was confirmed by the quantitative assessment when all patients were considered (Fig. 4b,c). When three imaging habitats were computed (K = 3), median dice similarity coefficient (DSC) for selected reproducible features was 0.94 and when all features were integrated, the median DSC was 0.78. Similarly, when five habitats were computed, selected features resulted in a higher median DSC than when all features were used, with a median DSC of 0.86 and 0.57, respectively.

## Discussion

Recent studies suggest using imaging habitats obtained from voxel-wise radiomics features of CT images improves quantification of tumor heterogeneity, and correlates better with the tumor biology and clinical outcomes^8,9^. However, to date the reproducibility of voxel-wise radiomics remains largely unknown. This study reports a comprehensive reproducibility analysis of voxel-wise radiomic features using CT scans from 500 lung cancer patients, image perturbations and different feature extraction parameters, providing reference values that could be used to guide the design of future studies.

We found that voxel-wise (3D) radiomic features of tumors were less reproducible than the conventional radiomics features with median CCCs of 0.63 and 0.96, respectively. These results highlight the importance of performing voxel-wise feature reproducibility assessment even in databases, where the 1D feature reproducibility has been previously quantified.

The reproducibility of voxel-wise features was also studied in perturbed images instead of the test–retest data, which are often not available in a clinical setting. Image perturbations have been previously applied in robustness assessment of conventional radiomics features^15^, however with contour perturbations. Using unperturbed contours, translated onto retest images, we observed that a single perturbed image with a lower magnitude of perturbations than previously published was able to mimic the retest scan. The perturbed scenario was able to reproduce the test–retest repeatability and to detect the most reproducible voxel-wise features.

The repeatable and reproducible voxel-wise features identified in this study could be divided in three structurally different groups, containing the following voxel-wise features: (1) joint entropy, sum entropy, difference entropy and Imc2 (2) maximum probability with joint energy, and (3) Idn, Idmn and Imc1. Most of these features characterize texture heterogeneity. Interestingly, features with low reproducibility describe the linear relationship between image intensity values (e.g., correlation), suggesting that the linear descriptors are the most affected by small changes in evaluated images, possibly due to high heterogeneity of the evaluated tissue. In previous publications, apart from energy and entropy measures, homogeneity and contrast were used to compute CT imaging habitats^8,10^. We have shown that the latter were not reproducible in the studied cohort.

Our results confirm that the voxel-wise features provide structurally different information, which could potentially provide information about biologically relevant tumor phenotypes. The biological meaning of radiomics is increasingly attracting attention, with different emerging approaches available, including genomic correlates, local microscopic pathologic image textures, and macroscopic histopathologic marker expression^16,17^. Most of the studies however, focus on multi-parametric MRI rather than CT imaging. Nevertheless, CT scans are by far the most extensively used imaging technique in clinical practice so the application of voxel-wise radiomics in unravelling the spatial distribution of tumor phenotypes from CT imaging opens up a new paradigm in the radiology field.

We acknowledge that our study carries some limitations. First of all, although we analyzed feature repeatability using perturbations across different datasets, we used a publicly available CT dataset with 30 patients (acquired using a single scanner type) to provide a test–retest habitats validation. Secondly, segmentation is a well-known source of variability in tumor volume assessment and consequently for the extracted radiomics features. In this study, we used a single contour to evaluate feature reproducibility and assure the voxel correspondence between test–retest scans. In the clinical scenario, the analyzed tumor volume can be assessed differently, which could affect the voxel feature values calculated at the tumor periphery. Moreover, we acknowledge that different unsupervised techniques as those used here could have been used to calculate voxel-by-voxel radiomics features, as for example unsupervised deep learning methods, e.g., deep autoencoders^18^. However, to our knowledge there is not a consensus on which architecture should be used to build deep unsupervised representations, while radiomics features based on the distribution of CT intensities and texture in voxel neighborhoods are now a reference standard in the community^19^. Exploring different deep architectures for feature calculation prior to habitat robustness assessment goes beyond the scope of this paper. Nonetheless, we acknowledge that this is an interesting and potentially useful research focus, which we plan to investigate in the future. Finally, we acknowledge that the robustness rankings presented in this paper apply to the specific case of lung cancer CT imaging, so that different radiomics features may be ranked as “robust” for habitat computation when other imaging modalities (e.g., MRI) or anatomical contexts (e.g., liver) are considered. Nonetheless, we point out that the same methodological evaluation framework presented here could be applied to assess robustness even in those cases. In future investigation, we will apply the evaluation framework presented in this paper to such other cases (e.g., liver MRI), with the aim of informing scientists working in the radiomics field.

In conclusion, imaging habitats computed using robust radiomics features are more stable than those computed using all features in test–retest CTs from the same patient. Voxel-wise radiomics features are more sensitive to changes in test–retest images than conventional radiomics texture features. The reproducibility of voxel-wise features can be studied using perturbed images, should retest images be unavailable. Moreover, we observed that the highly reproducible features provide additional spatial information, which could potentially reveal information on different biologically relevant tumor phenotypes. The voxel-wise feature selection methodology presented here can be applied for future robust imaging habitat computation. This study lays the foundation for the identification of reliable and informative voxel-wise features to discover previously unidentified tumor heterogeneity, with the aim of designing accurate biomarkers for diagnosis and prediction in precision medicine.

## Methods

### Patient cohort

Three different datasets with lung cancer patient CTs were investigated. Firstly, the RIDER lung CT open-access dataset was used in this study^15^, which is available to download on a TCIA platform https://wiki.cancerimagingarchive.net/. It consists of thoracic unenhanced test–retest CT scan pairs, acquired 15 minutes apart. Although data from 32 patients were downloaded, 2 patients were excluded due to incomplete data (image or segmentation missing). This data was the ‘ground-truth’ for validating habitats robustness.

Moreover, the Radiomics-NSCLC dataset was downloaded from the publicly available TCIA platform. 422 thoracic CT images with tumor segmentations were processed in this study. Lastly, we used a dataset from our institution (VHIO), which consists of 60 unenhanced thoracic CT scans with tumor segmentations.

### Image processing

Images and segmentations were converted from DICOM to NRRD. DCM2NIIX was used for image conversion^20^ (https://github.com/rordenlab/dcm2niix), while ITK-DCMQI (https://github.com/QIICR/ITK-dcmqi) toolbox was utilized for converting segmentations. To maintain voxel-voxel correspondence, only one segmentation was analyzed per CT pair. Test–retest pairs were registered using automatic rigid registration (6 DoF) using Elastix (https://elastix.lumc.nl/). For some patients, automatic registration failed and a manual adjustment was applied based on anatomical landmarks. An example of a registration result is shown in Supplementary Document SD1.

All images and segmentations were resampled to 1 mm × 1 mm × 1 mm voxel size using image resampling function from PyRadiomics^19^, which uses the SimpleITK library^21,22^. B-spline interpolation was used in CT images and the nearest neighbor interpolation was used in segmentations to avoid partial volume effects. Images were cropped to the bounding box defined by the segmentation extended by the default padding distance, necessary to compute some of the texture features. A summary of the image processing parameters is shown in Supplementary Document SD2 and is reported in the compliance with the Image Biomarker Standardization Initiative (IBSI) reporting guidelines^23^.

### Image perturbations

Test–retest imaging is recommended to assess feature reproducibility in a clinical setting; however, it is not always available especially when studying data retrospectively. In this study, we assessed reproducibility by perturbing test images using Medical Image Radiomics Processor (MIRP) available online (https://github.com/oncoray/mirp). MIRP was recently shown to be capable of generating image perturbation chains for accessing conventional radiomics feature robustness in lung and head and neck patient CT images^15^.

To reproduce the studied test–retest scenario, we considered three image perturbations: noise addition (N), translation (T) and rotation (R). In brief, noise addition perturbs image intensities by adding random noise that was drawn from a normal distribution with a mean of 0 and a standard deviation equal to the estimated standard deviation of the noise present in the image. Translation perturbs the image by affine transformation that shifts the image by a specified fraction (η) of the isotropic voxel spacing along the x, y and z axis. Finally, rotation perturbs the image with affine transformation that rotates the image in the axial (x, y) plane, i.e., around the z-axis, for a specified angle (θ).

Different perturbation scenarios were studied. A perturbation chain consists of different permutations of selected translation fractions and angles. We also explored them using a single perturbation image selected from the perturbation chain and found that it was able to more accurately reproduce the test–retest imaging scenario. Details on image perturbation parameters and scenario selection are provided in Supplementary Document SD3 and SD10.

### Voxel-wise feature extraction

In standard radiomics approach, Gray Level Co-occurrence Matrix (GLCM) features can be extracted in 2D, 2.5D or 3D tumor space. However, only a single value from a lesion is generated through averaging, direction or slice merging^23^, resulting in 1D representation of the analyzed feature.

Voxel-wise (3D) texture features were extracted from each of three analyzed images (test/retest/perturbed) of 30 studied patients. Twenty-four GLCM features were calculated from each segmented lesion using the open-source PyRadiomics Python package (https://github.com/AIM-Harvard/pyradiomics/). The maximal correlation coefficient (MCC) feature was excluded from the analysis due to the presence of missing values in most of the studied cases. Different combinations of feature extraction parameters i.e., fixed bin size (B) and kernel radius (R) were explored in test–retest data: B 12/25 HU and R 1/3/5 mm. Note, that in PyRadiomics the actual size of the kernel size is: 2 × R + 1. 9’360 3D feature maps were computed from the RIDER dataset.

Standardization of radiomics features is a common requirement for many machine learning models. In this study, the features were normalized to the range from 0 to 1, as test–retest CT scans from the same patient and lesion were compared for the reproducibility evaluation. An example of extracted features is provided in Supplementary Document SD5.

### Repeatability and reproducibility analysis

The repeatability of voxel-wise features was assessed in test–retest and test-perturbation image pairs. Perturbed images were used to create “synthetic retest” images. The reproducibility of voxel-wise radiomics features was analyzed between features computed using different feature extraction parameters (bin size and kernel radius). Repeatability and reproducibility were quantified using similarity measures frequently used to compare image quality: PCORR, (CCC^24^ and SSIM^25^. PCORR provides a measure of linear covariation between test–retest 3D feature maps, whereas CCC specifies the reliability based on both the linear covariation and the degree of correspondence between the test–retest features. In other words, the CCC correlation line passes through the origin and has a slope of 1 and therefore can be considered more sensitive than the PCORR. Another common reproducibility measure is the intra-class correlation (ICC)^26^, which can be computed in a number of paired data sets, whereas CCC is only computed in two datasets (e.g., test and retest). Since it is nearly identical to CCC^27^ and we compared between two paired datasets, we did not compute the ICC. Instead, we used another measure, SSIM, to capture the effect of distortions in structural information, which could be caused for instance by different noise levels in test–retest scans.

3D features variability is not only affected by the image quality, but also by the alignment of corresponding voxels, which could be subjected to the residual registration error. Therefore, we proposed to use the gamma index (Γ-index) which combines the feature difference and the distance to agreement into a single measure and can identify repeatable and reproducible features, which were not identified with CCC, PCORR or SSIM (see more information in Supplementary Document SD6). The gamma evaluation is routinely used in radiotherapy quality assurance to compare computed and measured inhomogeneous dose distributions^28,29^.

### Robustness of imaging habitats

Finally, we demonstrated the effect of selecting repeatable and reproducible features on imaging habitat computation. To eliminate redundant features, Principal Component analysis (PCA) was applied to select the most informative components from (i) all voxel-wise features and (ii) a subset of features identified as repeatable and reproducible. Five components were used to represent >93% of the variance from both feature groups in all 30 patients. Principal components were clustered using K-means (K = 3 and K = 5) to obtain imaging habitats. The robustness of imaging habitats computed from different features in test–retest data was quantified with the DSC.

## Supplementary Information

Supplementary Information.

## Data Availability

The “RIDER Lung CT” and “Radiomics-NSCLC” CT image data are on the National Biomedical Imaging Archive (TCIA) site (https://imaging.nci.nih.gov/ncia) and were de-identified in compliance with HIPPA requirement for sharing data. VHIO CT dataset cannot be made publicly available due to Spanish data protection laws. These data could be potentially obtained from the corresponding author on a reasonable (academic) request, and pending approval by the local ethics committee. 3D features were extracted using an open-source python package PyRadiomics https://github.com/AIM-Harvard/pyradiomics/. Gamma index was computed using an open-source image processing platform OpenREGGUI (https://openreggui.org/).
