# Supplementary material for: Robust imaging habitat computation using voxel-wise radiomics features
Source: Sci Rep. 2021 Oct 11;11:20133. doi: 10.1038/s41598-021-99701-2 (PMC8505612; doi:10.1038/s41598-021-99701-2)
Supplement: Supplementary file 1 — Supplementary Information. [file 41598_2021_99701_MOESM1_ESM.docx]

**SUPPLEMENTARY DOCUMENT (SD)**

**Title:** Robust imaging habitat computation using voxel-wise radiomics features

**Authors:** Kinga Bernatowicz, Francesco Grussu, Marta Ligero, Alonso Garcia, Eric Delgado, and Raquel Perez-Lopez

SD1 Figure: Test-retest images before and after registration. Notice how the original segmentation (green) was aligned to the tumor volume after registration.


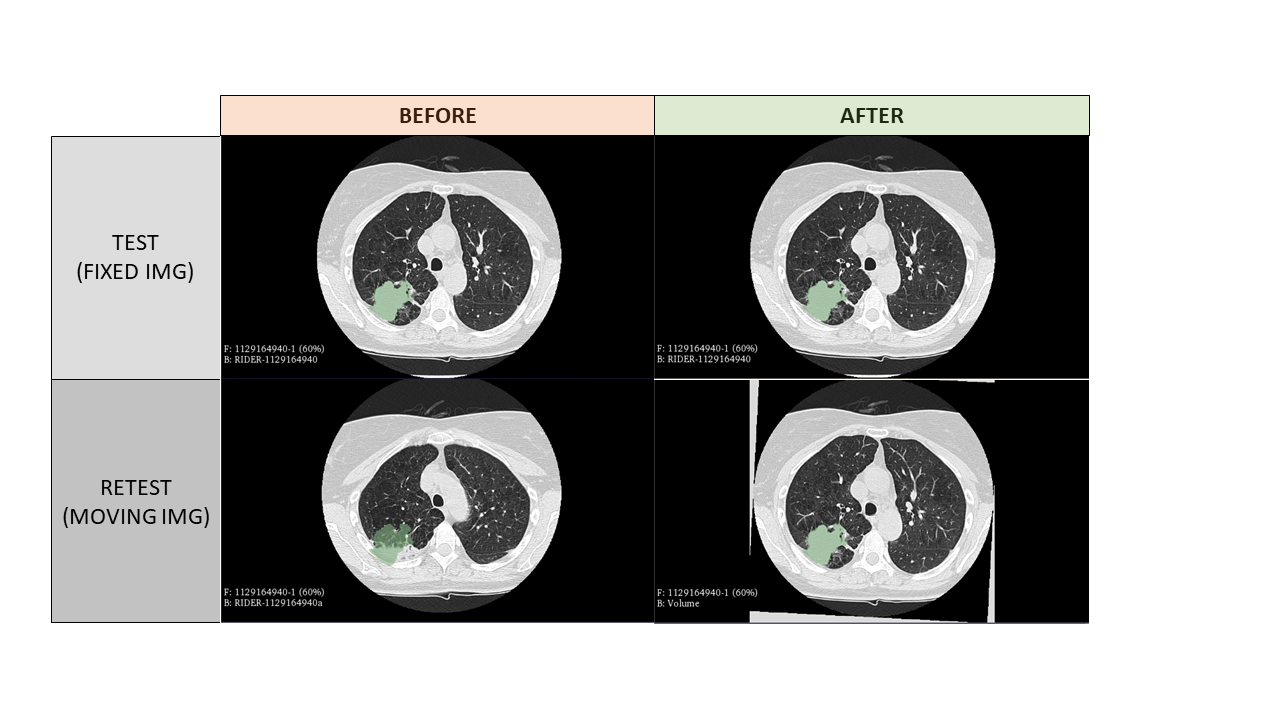


SD2 Table: Image processing and feature extraction parameters.

| **Interpolation** | Resampled voxel spacing (mm) | 1 |
| --- | --- | --- |
|  | Interpolation method | B-spline |
|  | Intensity rounding | No |
|  | ROI interpolation method | Nearest neighbor |
| **Re-segmentation** |  | No |
| **Feature extraction** | Fixed bin size (HU) | 12 / 25 |
|  | Kernel radius (mm) | 1 / 3 / 5 |

SD3 Text and Figure: Image perturbation scenarios and parameters

Image perturbation chain (PCH) consists of different permutations of selected translation fractions and angles. Two different sets of parameters were studied in scenario 1 and 2 of the perturbation chain. We also explored utilizing single perturbation (SP) image selected from the PCH scenario 2, which resulted in most similar image to retest scenario, according to the concordance correlation coefficient as shown in Figure below. The bar plot represents the mean CCC value and standard deviation.

*Scenario 1*: PCH based on a publication Zwanenburg et al.

Noise addition, translation, rotation (NTR, n = 32)

• noise addition: 1 repetition

• translation: 𝜂 = {0.25, 0.75}

• rotation: θ = {-6°, -2°, 2°, 6°}

*Scenario 2*: PCH with smaller translation and rotation

Noise addition, translation, rotation (NTR, n = 16)

• noise addition: 1 repetition

• translation: 𝜂 = {0, 0.1}

• rotation: θ = {0.5°}

*Scenario 3*: SP

Noise addition, translation, rotation (NTR, n = 1)

• noise addition: 1 repetition

• translation: 𝜂 = {0.1}

• rotation: θ = {0.5°}


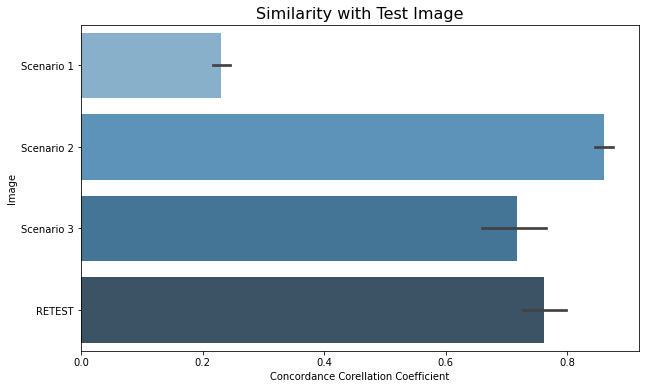


SD4 Table: Pixel spacing in test-retest DICOM images and lesion volume of studied patients’ cohort. Figure shows the range (whiskers), median value and Q1-Q3 interquartile range (box).


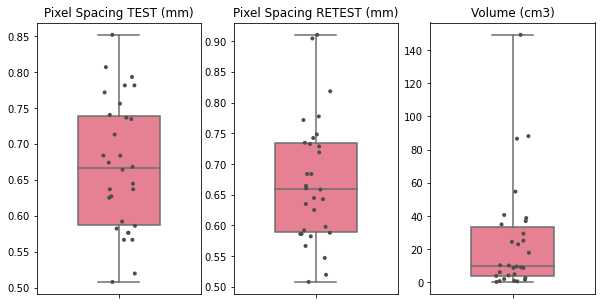


SD5 Figure: Axial section of the voxel-wise features from test images of two different patients.


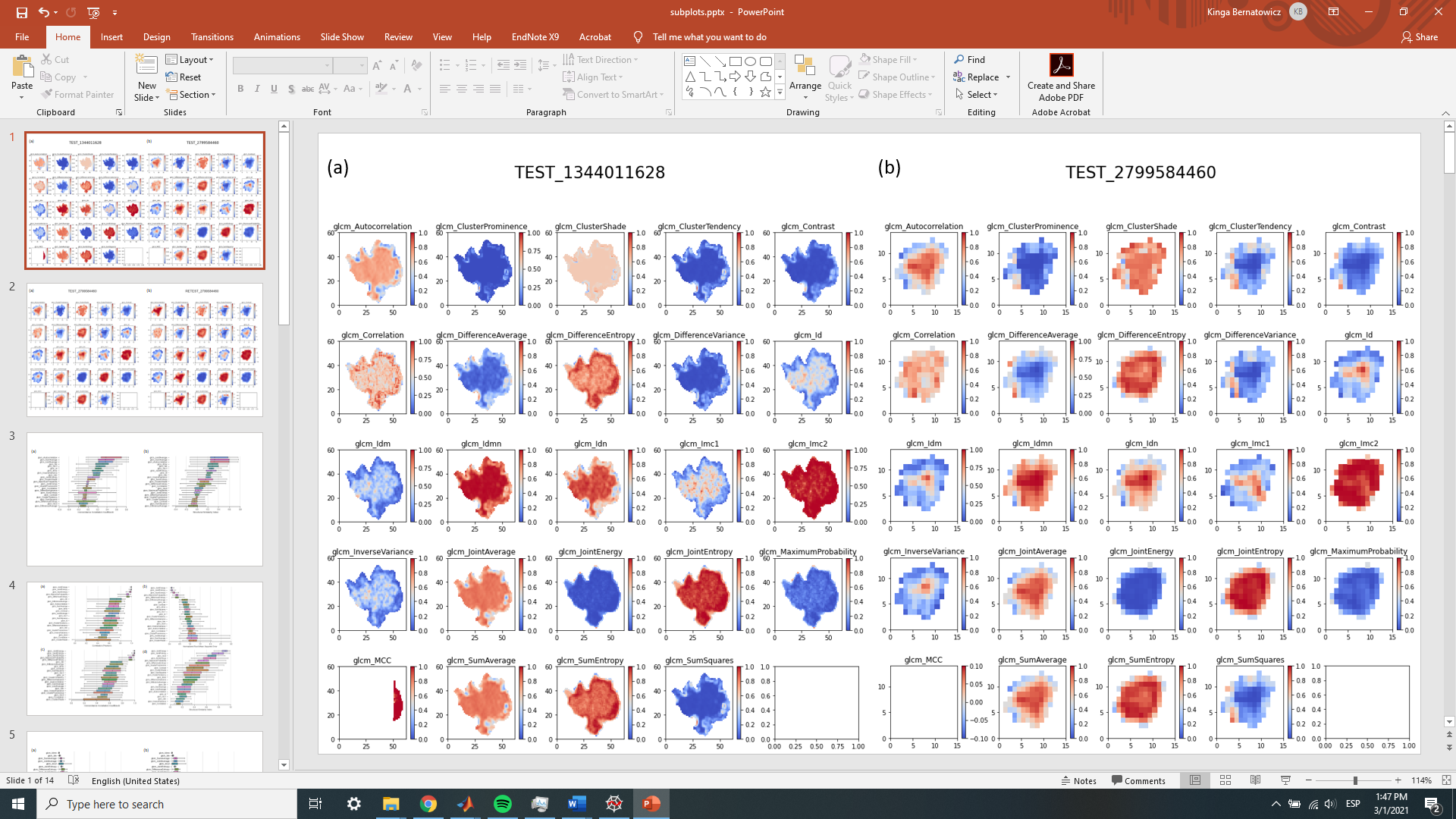


SD6 Text, formula and figure: Gamma index definition and example in feature reproducibility evaluation.

In space composed of feature and spatial coordinates, the gamma acceptance criteria form an ellipsoid surface around the reference point. The acceptance criteria are defined as the maximum feature value in percent ($F_{max}$) and distance to agreement ($\mathrm{DTA}$) in mm and are effectively scaling factors. When the evaluated feature point passes through the ellipsoid, it passes the gamma acceptance test for the reference point.

In this work points are defined in the voxel center; reference corresponds to the test feature and evaluated feature is either retest or perturbed feature. Therefore, gamma values (**Γ**) were computed in each point/voxel using:

$$\boldsymbol{\Gamma}(r_{ref},r_{eval})=\sqrt{\frac{{\Delta r}^{2}}{\mathrm{DTA}}+\frac{{\Delta F}^{2}}{F_{max}}}$$

Where ${\Delta r= |r}_{ref}-r_{eval}|$ denotes the distance between analyzed voxels and in 3D coordinate system is computed as

$\sqrt{{{(x}_{ref}-x_{eval})}^{2}+{{(y}_{ref}-y_{eval})}^{2}+{{(z}_{ref}-z_{eval})}^{2}}$

and $\Delta F= {|F}_{ref}(r_{ref})-F_{eval}(r_{eval})|$ is the difference in the voxel-wise feature values as depicted in Figure below (in 2D spatial coordinates and feature domain).


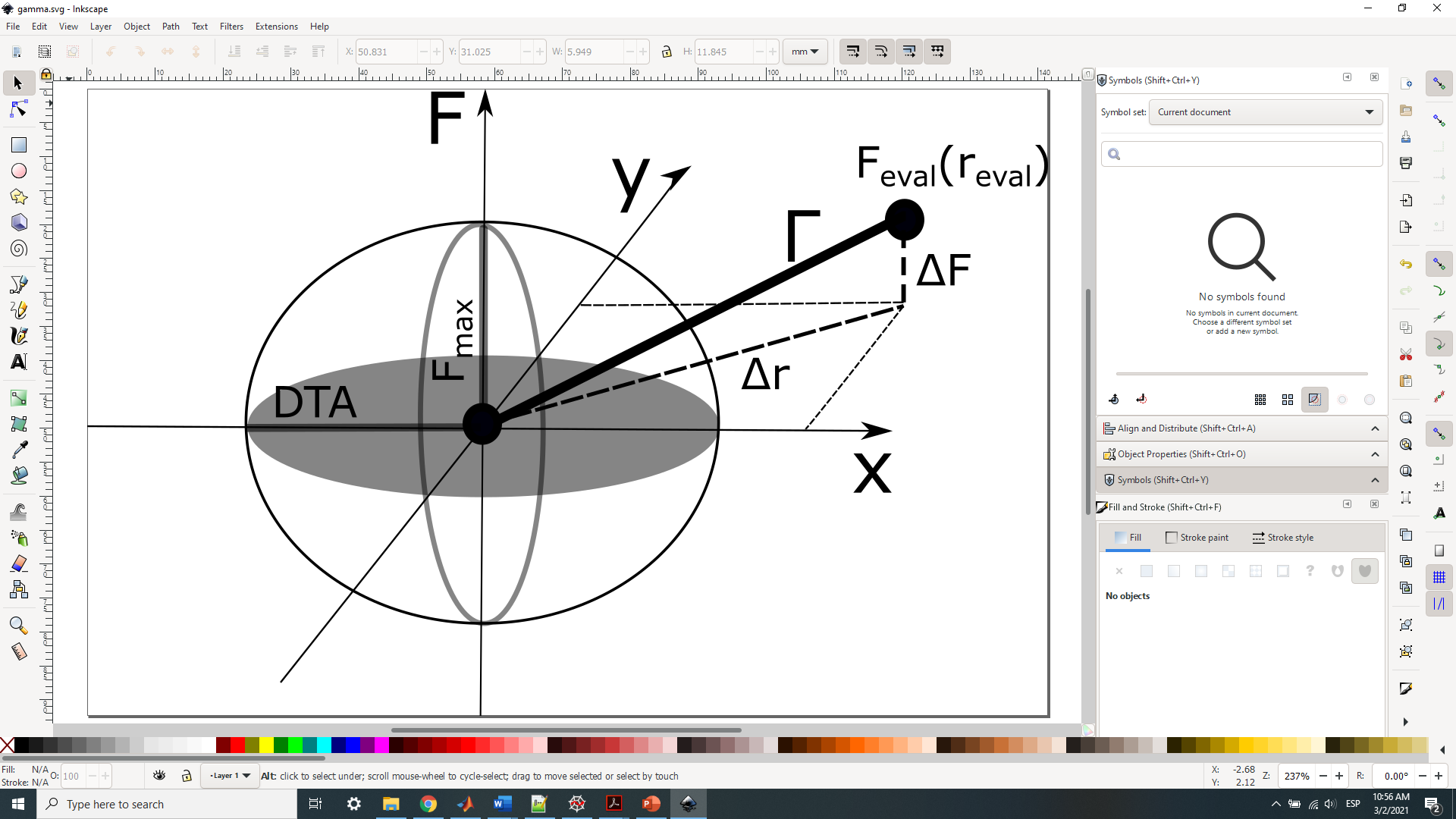


After computing gamma values for each voxel in the distribution, the number of voxels meeting predefined criteria (DTA and F_max_) can be computed by summing up all voxels with Γ < 1. Example of gamma values computation at different acceptance criteria and comparison with other reproducibility metrics is shown in Figure below. The following short names were used: Concordance Correlation Coefficient (CCC), Pearson’s Correlation (PCORR) and Structural Similarity Index (SSIM), Informational Measure of Correlation (Imc1).


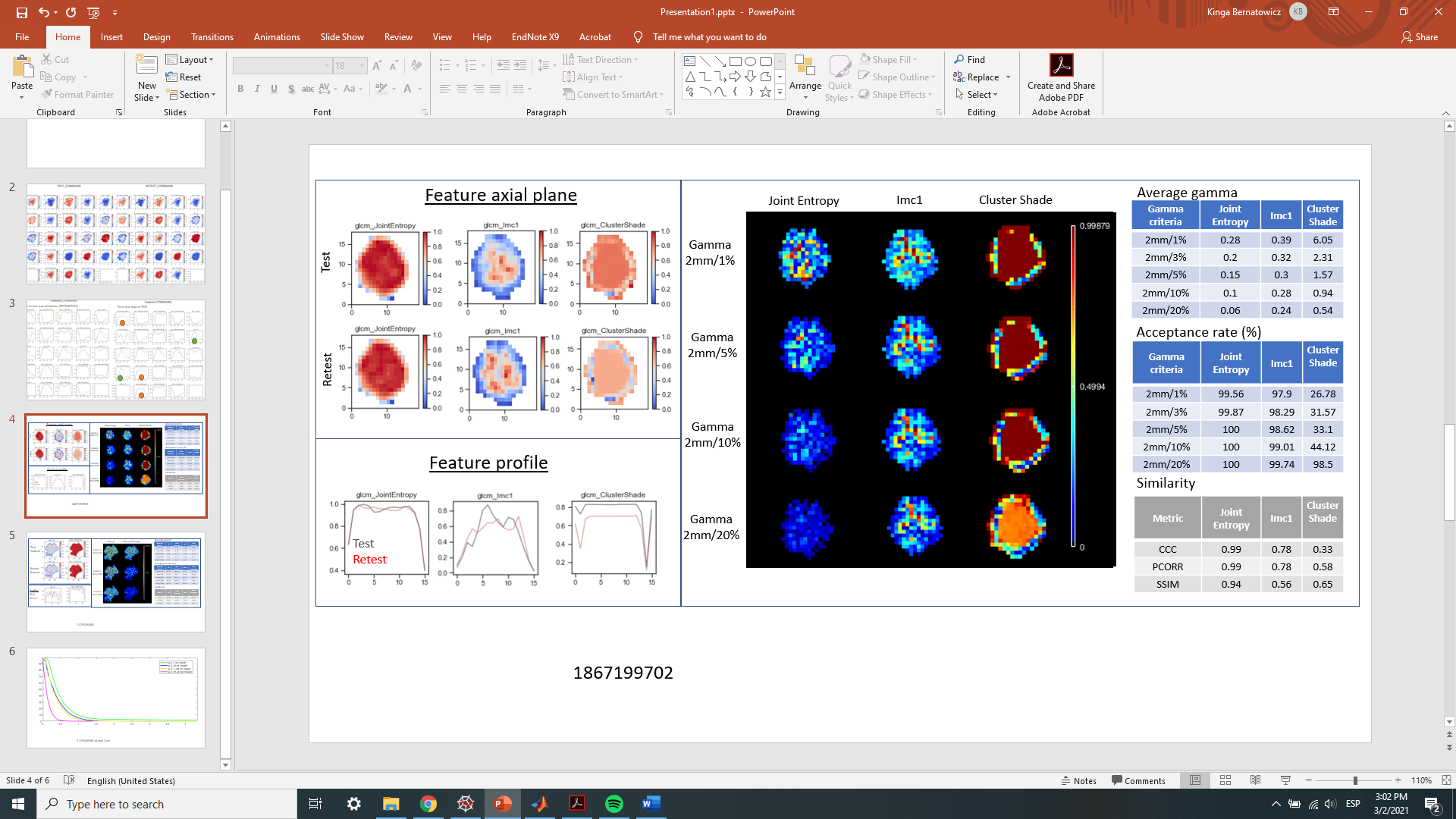


SD7 Figure: Voxel-wise feature repeatability – PCORR and SSIM computed in the RIDER dataset. Boxplots representing repeatability of features in (a, b) test-retest data and (c, d) test-perturbed CTs. Boxplots are colored by the feature and most repeatable features are arranged on the top of the plot. Note that the most repeatable features describing energy and entropy are very similar independent on the evaluation metrics.


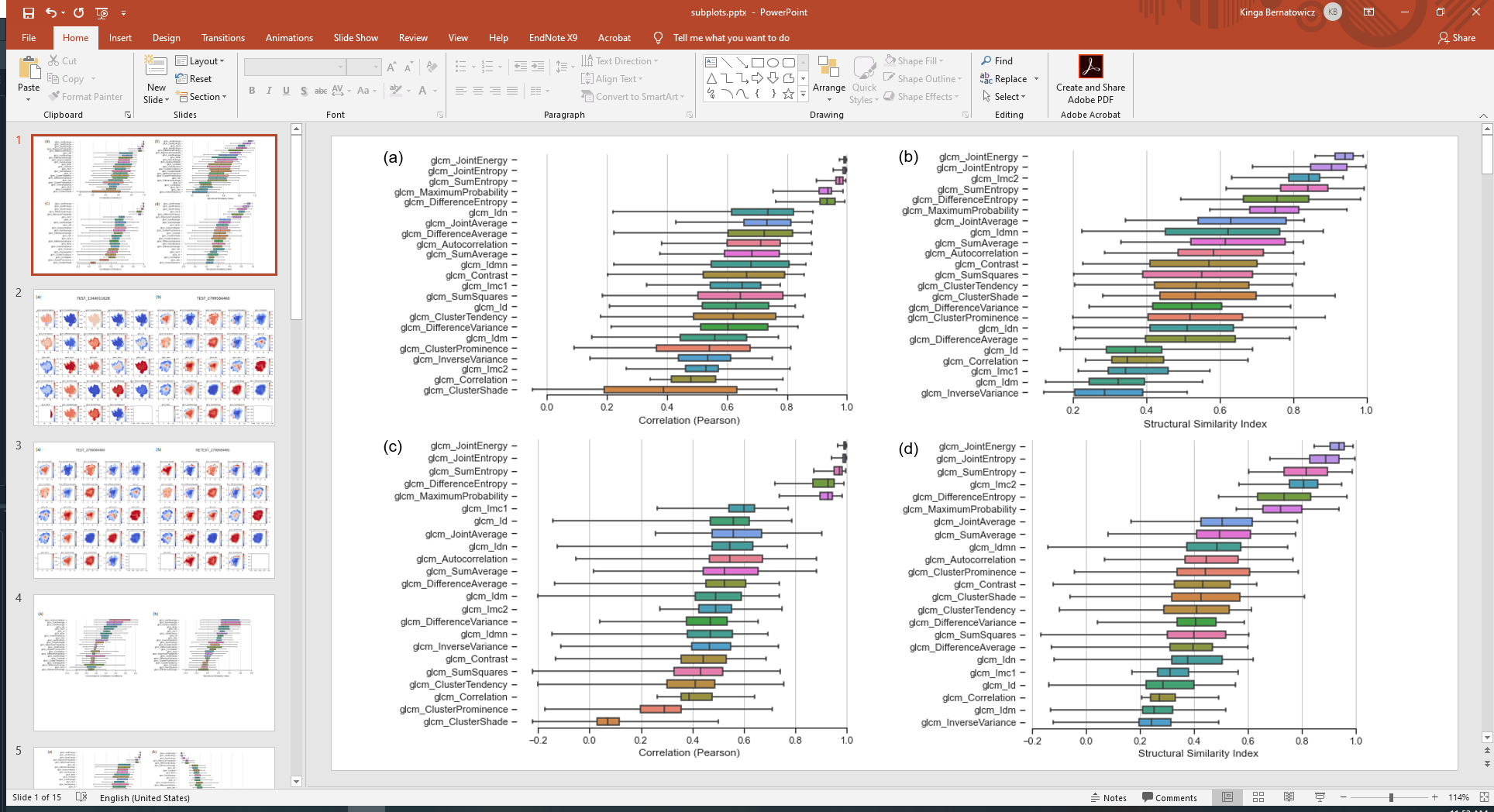


SD8 Text and Figure: Non-redundancy of voxel-wise features computed in the RIDER dataset.

Ultimately, robust voxel-wise features could be used to aggregate similar tumor subregions that represent imaging habitats. Therefore, it is crucial to assess whether voxel-wise features contain more information than could be obtained directly from voxel-wise Hounsfield unit (HU) values, whether features provide non-redundant spatial information and whether this information is present in a subset of robust features. The non-redundancy of voxel-wise HU values (from test CT image) and features extracted from the test CT was assessed using SSIM and PCORR.

Median SSIM and PCORR from voxel-wise features extracted from the test CTs are presented as a heatmap in Figure below. Additionally, test CT values (HU) were compared with the voxel-wise features and were not correlated nor structurally similar with any voxel-wise feature (SSIM and PCORR values close to 0). This is interesting, as different voxel-wise features provide additional spatial information, which could potentially inform about biologically relevant tumor phenotypes.

Regarding the most repeatable and reproducible features identified earlier based on CCC and Γ-index and across different feature extraction parameters (joint energy, joint entropy, sum entropy, maximum probability, difference entropy, Imc1, Imc2 and Idmn), three structurally different groups can be distinguished based on SSIM: (1) all 3 entropy-related features and Imc2 (2) maximum probability with joint energy, and (3) Idmn and Imc1, as shown in Figure below (a). These groups were confirmed with a positive PCORR within the groups (b). Additionally, a negative correlation between groups 1 and 2 was found (PCORR ≈ -1).

Figure: Similarity of CT values and voxel-wise features. (a) Median Structural Similarity Index and (b) Pearson’s Correlation Coefficient. Features were extracted from test scans of all studied patients using R = 1 and B = 12 settings.


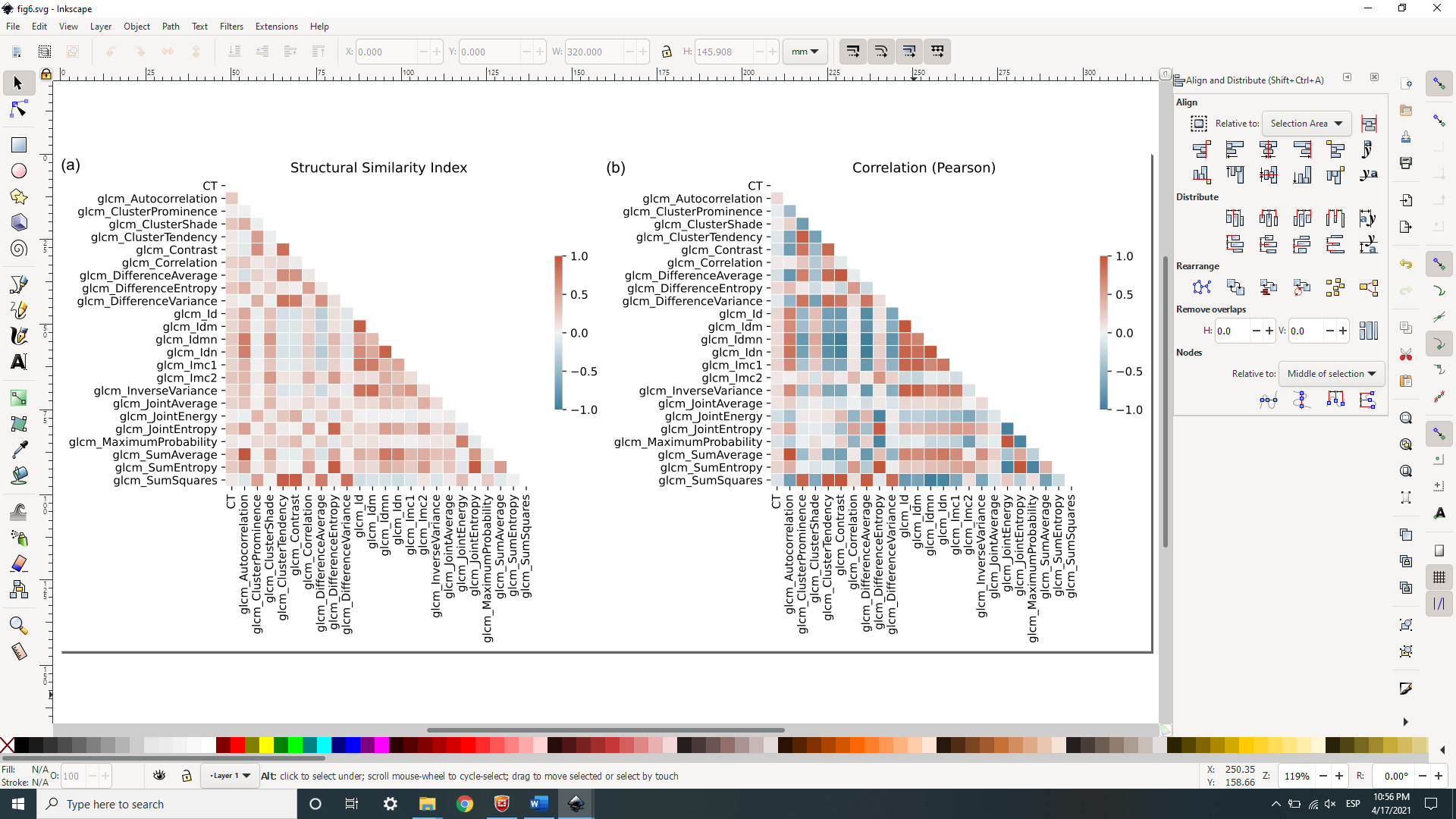


SD9 Text, Table and Figure: Correlation of repeatability with lesion volume and imaging parameters computed from the RIDER dataset.


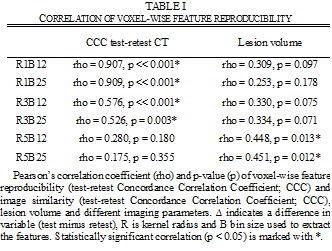


The reproducibility of voxel-wise features was evaluated in all 30 patients (see Figure below). Only 3 patients had median CCC higher than 0.8 and most patients had a large CCC range, confirming large differences in reproducibility between features independently on selected patient.

Interestingly, a significant correlation (rho = 0.91, p << 0.001) was observed when comparing test-retest feature similarity with originating test-retest image similarity for features extracted with R = 1 and B = 12 (Figure), but this relationship was not significant when R = 5 (Table). Low but significant correlation with lesion volume was observed for features extracted with the largest kernel radius and no correlation with acquired pixel spacing difference was observed, see Table.

Figure: Voxel-wise feature reproducibility (R1B12) in studied lung cancer patients (a). Comparison of test-retest CT image similarity (Concordance Correlation Coeffient_x) with voxel-wise feature similarity computed on those images (Concordance Correlation Coefficient_y) (b). Different points correspond to studied patients.


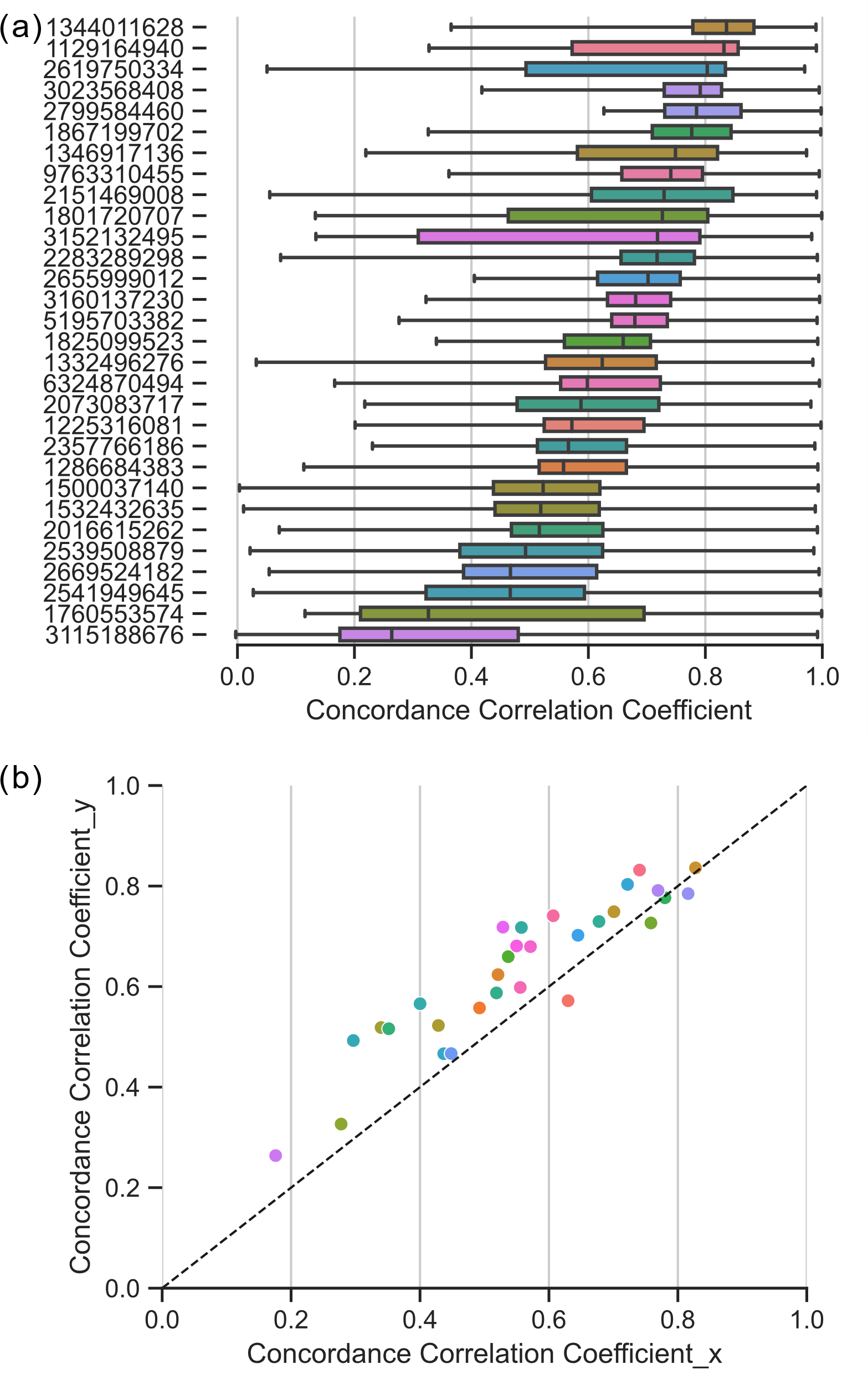


SD10 Figure: Voxel-wise feature repeatability (CCC) computed in (a) the NSCLC-Radiomics imaging database and (b) VHIO dataset acquired at the Vall d’Hebron Hospital in Barcelona. Perturbations were performed using NTR scenario (see SD3), where translation parameter used in RIDER dataset was scaled to the median voxel size of NSCLC-Radiomics and VHIO datasets (𝜂 = {0.05}). Boxplots representing repeatability of features in test-perturbed CTs. Boxplots are colored by the feature and most repeatable features are arranged on the top of the plot. Note that the ranking of most repeatable features remains the similar to the RIDER dataset evaluation - features describing energy and entropy are on the top of the plot.


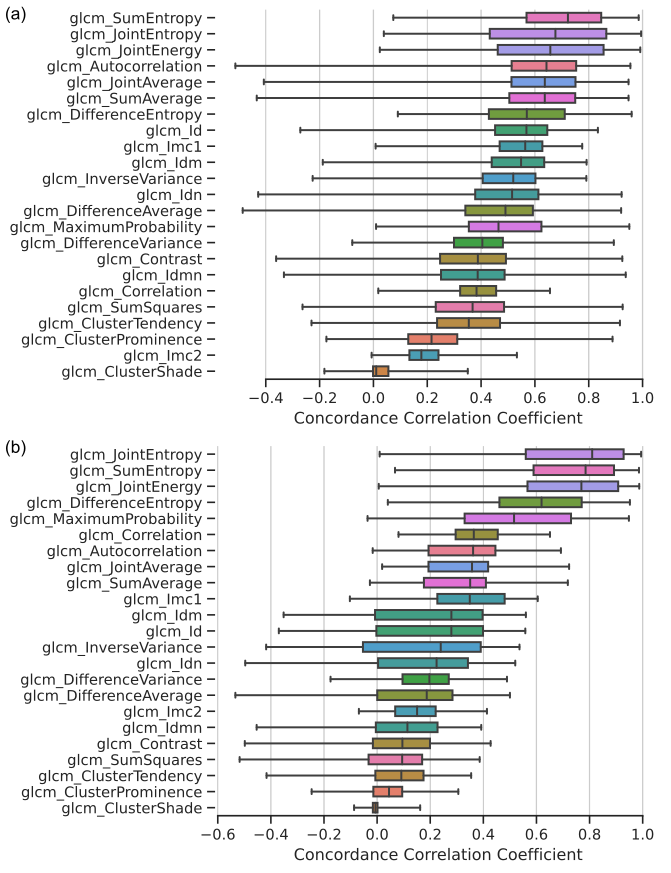
.
